# CTCF loss induces giant lamellar bodies in Purkinje cell dendrites

**DOI:** 10.1186/s40478-022-01478-6

**Published:** 2022-11-29

**Authors:** Teruyoshi Hirayama, Yuuki Kadooka, Etsuko Tarusawa, Sei Saitoh, Hisako Nakayama, Natsumi Hoshino, Soichiro Nakama, Takahiro Fukuishi, Yudai Kawanishi, Hiroki Umeshima, Koichi Tomita, Yumiko Yoshimura, Niels Galjart, Kouichi Hashimoto, Nobuhiko Ohno, Takeshi Yagi

**Affiliations:** 1 KOKORO-Biology Group, Laboratories for Integrated Biology, Graduate School of Frontier Biosciences, Osaka University, Suita, 565-0871 Japan; 2 Department of Anatomy and Developmental Neurobiology, Tokushima University Graduate School of Medical Sciences, 3-18-15 Kuramoto-cho, Tokushima, 770-8503 Japan; 3 Section of Electron Microscopy, Supportive Center for Brain Research, National Institute for Physiological Sciences, Okazaki, 444-8787 Japan; 4 Department of Anatomy II and Cell Biology, Fujita Health University School of Medicine, 1-98 Dengakubo, Kutsukake-cho, Toyoake, 470-1192 Japan; 5 Department of Physiology, Division of Neurophysiology, School of Medicine, Tokyo Women’s Medical University, Tokyo, 162-8666 Japan; 6 Department of Neurophysiology, Graduate School of Biomedical and Health Sciences, Hiroshima University, 1-2-3 Kasumi, Minami-ku, Hiroshima, 734-8551 Japan; 7 Section of Visual Information Processing, National Institute for Physiological Sciences, National Institutes of Natural Sciences, Okazaki, Aichi 444-8585 Japan; 8 Department of Physiological Sciences, The Graduate University for Advanced Studies, Okazaki, Aichi 444-8585 Japan; 9 Department of Cell Biology, Erasmus University Medical Center, P.O. Box 2040, 3000 CA Rotterdam, The Netherlands; 10 Division of Ultrastructural Research, National Institute for Physiological Sciences, Okazaki, 444-8585 Japan; 11 Department of Anatomy, Division of Histology and Cell Biology, Jichi Medical University, Shimotsuke, 329-0498 Japan

**Keywords:** CCCTC-binding factor, Giant lamellar body, Purkinje cell, Neurodegeneration, Motor dysfunction

## Abstract

CCCTC-binding factor (CTCF) has a key role in higher-order chromatin architecture that is important for establishing and maintaining cell identity by controlling gene expression. In the mature cerebellum, CTCF is highly expressed in Purkinje cells (PCs) as compared with other cerebellar neurons. The cerebellum plays an important role in motor function by regulating PCs, which are the sole output neurons, and defects in PCs cause motor dysfunction. However, the role of CTCF in PCs has not yet been explored. Here we found that the absence of CTCF in mouse PCs led to progressive motor dysfunction and abnormal dendritic morphology in those cells, which included dendritic self-avoidance defects and a proximal shift in the climbing fibre innervation territory on PC dendrites. Furthermore, we found the peculiar lamellar structures known as “giant lamellar bodies” (GLBs), which have been reported in PCs of patients with Werdnig-Hoffman disease, 13q deletion syndrome, and Krabbe disease. GLBs are localized to PC dendrites and are assumed to be associated with neurodegeneration. They have been noted, however, only in case reports following autopsy, and reports of their existence have been very limited. Here we show that GLBs were reproducibly formed in PC dendrites of a mouse model in which CTCF was deleted. GLBs were not noted in PC dendrites at infancy but instead developed over time. In conjunction with GLB development in PC dendrites, the endoplasmic reticulum was almost absent around the nuclei, the mitochondria were markedly swollen and their cristae had decreased drastically, and almost all PCs eventually disappeared as severe motor deficits manifested. Our results revealed the important role of CTCF during normal development and in maintaining PCs and provide new insights into the molecular mechanism of GLB formation during neurodegenerative disease.

**Supplementary Information:**

The online version contains supplementary material available at 10.1186/s40478-022-01478-6.

## Introduction

Epigenetic modifications and higher-order chromatin architecture are important for establishing and maintaining cell identity by controlling gene expression [5, 30]. The ability of CTCF to bind DNA is affected by epigenetic modification of its recognition motif, and this factor has a key role in three-dimensional (3D) chromatin loop structures through CTCF-mediated chromatin interactions [9, 29, 39, 41]. 3D chromatin structures are thought to limit the interactions of regulatory elements with genes within the same chromatin loop while at the same time insulating them from interactions with other genomic regions. Dysregulation of these interactions causes various neurodevelopmental, psychiatric, and neurodegenerative disorders [4, 8, 43, 44].

CTCF is associated with several human diseases. The initial genetic study of *CTCF* mutations reported that four patients had different de novo mutations at the *CTCF* locus and exhibited intellectual disability, microcephaly, or growth retardation; in addition, two patients showed autistic behaviour [15]. More recently, the same and other groups have identified many individuals with variant mutations in the *CTCF* locus [1, 25, 31]. These individuals showed variable severity, ranging from mild developmental delay or a normal intelligence quotient to severe intellectual disability. In a study that used an integrated pathway-based approach to evaluate more than 10,000 individuals of European ancestry in conjunction with data from the Psychiatric Genomics Consortium Schizophrenia, certain mutations in the *CTCF* locus identified as a strong risk factor for schizophrenia [27].

The role of CTCF in the nervous system has also been demonstrated using conditional knockout (cKO) mice [7]. CTCF activities regulate the balance between neuroprogenitor proliferation and differentiation in the telencephalon and are necessary for the survival of excitatory and inhibitory neurons [6, 49]. In postmitotic cortical projection neurons, CTCF has an essential role in normal brain development, including dendrite arborization, dendritic-spine growth, and somatosensory barrel formation [24]. Deletion of CTCF in postmitotic neurons also results in defects in learning and memory abilities with impaired long-term potentiation and reduced spine density [28, 42]. In addition, deletion of CTCF induces hearing impairment, impaired nerve regeneration, and a neurobehavioural phenotype with overexpression of inflammation-related genes [33, 34, 40]. These results indicated that CTCF is essential for normal neural development and functional neural networks.

The cerebellum, in which PCs are the sole output neurons, plays an important role in motor regulation, and defects in PCs lead to motor dysfunction. In the mature cerebellum, CTCF is highly expressed in PCs as compared with other cerebellar neurons. In addition, deletion of clustered protocadherin (cPcdh) family proteins in PCs results in dendritic self-avoidance defects [26, 32]. The *cPcdh* family represents a major set of genes regulated by CTCF [24]. Thus, gene regulation by CTCF is expected to be important for normal PC development and maintenance. However, the role of CTCF in PCs has not yet been explored.

## Materials and methods

### Generation of glutamate receptor δ2 (*Grid2*)-Cre-dependent CTCF-deficient mice

The experimental procedures for animals were in accordance with the guide for the care and use of laboratory animals of the science council of Japan and were approved by the animal experiment committee of Osaka or Tokushima University. All mice were maintained under specific pathogen-free conditions. *Ctcf*-floxed mice, in which loxP sites flank exons 3–12, were described previously by Heath et al. [21]. Mice conditionally lacking CTCF (CTCF-cKO) in PCs were generated by breeding *Ctcf*-floxed mice with *Grid2*-Cre mice. *Grid2*-Cre mice were generated using a knock-in strategy, and Cre recombinase was predominantly expressed in PCs and in molecular layer interneurons at lower levels [50]. *Ctcf*;*Grid2*-Cre (+/fl; +/+) and *Ctcf*;*Grid2*-Cre (fl/fl; +/+) mice were used as controls.

### Histological analysis

Mice were deeply anaesthetized and transcardially perfused with phosphate-buffered saline (PBS) followed by 4% paraformaldehyde (PFA) in 0.1 M phosphate buffer (PB). Then, the brains were removed and postfixed in the same fixative for 2 h at 4 °C and subsequently cryoprotected in 30% sucrose in PB. Frozen sections, either 10 or 50 μm thick, were prepared on a microtome. Sections were washed with PBS and incubated for 1 h at room temperature in blocking buffer: 20% Block Ace (KAC Co., Ltd.), 5% normal goat serum (NGS), 0.1% Triton X-100, 0.1% azide in PBS. Then, the sections were incubated overnight with primary antibody in antibody dilution buffer (5% Block Ace, 5% NGS, 0.1% Triton X-100, 0.1% azide in PBS) at 4 °C. Sections were then washed with 0.1% Triton X-100 in PBS and incubated for 1 h with secondary antibody in antibody dilution buffer at room temperature. The antibodies used were as follows: anti-calbindin (1:500; Sigma-Aldrich), anti-CTCF (1:1000; Cell Signaling Technology), anti-VGluT2 (1:10,000; Millipore), anti-active caspase-3 (1:500; Cell Signaling Technology), anti-calnexin (1:500; Enzo), anti-KDEL (1:2000; MBL), and anti-IP3R (1:500; abcam). For haematoxylin and eosin (HE) staining, sections were stained with Mayer’s haematoxylin and eosin Y (Muto Pure Chemicals, Tokyo, Japan).

### In situ hybridization analysis

In situ hybridization was performed as described [24]. In brief, mice were deeply anaesthetized, and their brains were removed and embedded in OCT compound (Sakura Finetek, CA, USA) as quickly as possible and then frozen in isopentane cooled with liquid nitrogen. The frozen tissue was cut into 10-μm sections on a cryostat (CM1850, Leica Microsystems). Digoxigenin (DIG)-labelled RNA probes were synthesized from cDNA clones using DIG RNA Labelling Mix (Roche). In situ hybridization RNA probes were as follows: the fragments corresponding to + 1519 (5′-TTTGCTGATCAGACTGGCGT-3′) to + 2303 (5′-ACGGTTGTTCAGTCCCATCC-3′) of mouse *Galc* cDNA, + 33 (5′-GCAGGAAGATACGGTGCTGT-3′) to + 752 (5′-TCTCCCGACTGTCTGGATGA-3′) of mouse *Smn1* cDNA, + 399 (5′-CCCTGAAACCAACAAAAACA-3′) to + 1050 (5′-ATGATAATGCACCAGAGGTC-3′) of mouse *Pcdhα12* cDNA and + 318 (5′-CGTGAGCTTTAACATCTTGA-3′) to + 1291 (5′-GAAGGCCACAGATGGTGGAA-3′) of mouse *Pcdh γA7* cDNA.

### Behavioural assays

Gait analysis was carried out using a footprint test with a runway measuring 30 cm in length and 6 cm in width with walls that were 50 cm high [2]. Habituation was performed by allowing the mice to walk freely along the narrow runway before their footprints were collected. To collect the footprints, a new sheet of white Japanese calligraphy paper was placed on the floor of the runway. Just before the footprint test, the feet of the individual mouse were painted with nontoxic paint (orange or black). Then, the mice were gently held at the entrance of the runway and released. Footprints were analysed for print separation (distance between corresponding fore- and hindpaw prints), front and hind width (distance between left and right prints of the fore- and hindpaws), front- and hind-stride length (distance between each footprint), front and hind ratio (ratio of width/length of each footprint).

The open-field test was performed using IMAGE OF4 (O’Hara & Co., Ltd.), which consists of a white plastic square chamber (50 × 50 × 40 [H] cm) with a CCD camera on the ceiling. Locomotor activity was automatically measured by IMAGE OF4 software (O’Hara & Co., Ltd.) for 10 min at P50, and we analysed total walking distance.

For the walking initiation test, each mouse was placed in the middle of a square outlined by white plastic tape (21 × 21 cm) on the smooth black surface of a large tabletop. We measured the time it took each mouse to leave the square (to place all four paws outside of the tape). When the mouse did not leave the square within 60 s, we stopped the test.

For the beam test, we used a beam apparatus with a flat surface (width, 28 mm; length, 700 mm) resting 20 cm above the tabletop on two poles. A box with nesting material from the home cage was placed at one end of the beam to lure the mouse, which was placed at the opposite end of the beam, to the finish point. Before the test, each mouse was allowed to cross the beam two times. During the test, we measured the time it took each mouse to go from the start to the finish point. Two trials were averaged for each mouse. When the mouse did not reach the finish point within 60 s or fell from the beam, the time was set to 60 s.

The platform test was performed as described [14], with some modifications. In brief, each mouse was timed for how long it remained on an elevated, square platform (3 × 3 cm, 1 cm thick) with rounded edges. When the mouse remained on the platform for the entire test trial or fell from the platform, the time was calculated as a maximum score of 60 s.

For the pole test, a mouse was placed with its head upwards on top of a vertical metal rod (diameter, 8 mm; height, 70 cm), the surface of which had been covered by medical tape. We then measured the time the mouse took to descend to the floor, with a maximum duration of 120 s. If a mouse fell from the pole before reaching the floor, it was given the maximum score of 120 s for that trial. Two trial scores were averaged for each mouse.

The screen test was performed as described [14], with some modifications. In brief, each mouse was placed on top of an elevated (30 cm above the floor) wire mesh grid. Then, the screen was inverted by 180°, and we measured how long the mouse was able to remain upside down on the screen. If a mouse did not fall from the screen for the entire duration, it was given the maximum score of 90 s.

The rotarod test was performed as described [14], with some modifications. In brief, the test involved two conditions: a rotating rod with a constant speed (5 rpm for 60 s) and a rod that had an accelerating rotational speed (0–3 rpm for 2 min, with the rod accelerating during the first 60 s) using RRAC-3002 (O’Hara & Co., Ltd.). Mice at P56–62 and P175–182 were used. We conducted three sessions, every 3 days. Each session included two trials at a constant speed and two trials at an accelerating rotational speed with 10 min of rest between each trial.

### In vitro whole-cell patch-clamp recordings

Mice at P47–59 were deeply anesthetized by CO_2_ or isoflurane inhalation and were decapitated. The brains were quickly removed, and parasagittal slices with a thickness of 250 μm were prepared from the cerebellar vermis with a Vibratome slicer (VT1200S, Leica) in chilled normal artificial cerebrospinal fluid (ACSF) containing 125 mM NaCl, 2.5 mM KCl, 2 mM CaCl_2_, 1 mM MgSO_4_, 1.25 mM NaH_2_PO_4_, 26 mM NaHCO_3_, and 20 mM glucose, bubbled with 95% O_2_ and 5% CO_2_. Cerebellar slices were kept at 25 °C in normal ACSF. Whole-cell recordings were made from visually identified PCs using an upright microscope (BX50WI, Olympus). Normal ACSF supplemented with bicuculline (10 μM, Tocris) or picrotoxin (100 μM, Tocris) was used as a bath solution during recordings to block GABAergic transmission. The pipette solution consisted of 60 mM CsCl, 10 mM Cs _D_-gluconate, 20 mM TEA-Cl, 20 mM BAPTA, 4 mM MgCl_2_, 4 mM ATP, 0.4 mM GTP, and 30 mM HEPES (pH 7.3, adjusted with CsOH). The pipette solution also contained 0.5% neurobiotin (Vector labs) for post hoc visualization of the recorded cells. A glass pipette for stimulating climbing fibres was filled with normal ACSF. Its position was moved systematically around each PC soma. The number of climbing fibres innervating the recorded cell was estimated as the number of discrete EPSC steps during a gradually increasing stimulus intensity (0–90 V) at each stimulus location [35, 36]. To calculate the disparity ratio, the amplitudes of each CF-EPSCs in a given multiply innervated PC were measured and numbered in amplitudes (A_1_, A_2_, …, A_N_, N ≧ 2, A_N_ is the largest one). Then, the disparity ratio was obtained from the following formula: Disparity ratio = (A_1_/A_N_ + A_2_/A_N_ + ⋯ + A_N−1_/A_N_)/(N − 1). If all CFs in a given PC evoke EPSCs with the same amplitude, the disparity ratio will be 1. Conversely, if the differences between the largest (A_N_) and other smaller CF-EPSC amplitudes are large, the disparity ratio will be small [18]. All experiments were examined and analysed by investigators who were blind to the mouse genotypes. All data were recorded at 32 °C with an EPC10 patch-clamp amplifier with Patch Master software (HEKA Elektronik). Offline data analyses were performed using Fit Master software (HEKA Elektronik), Excel (Microsoft), and IGOR Pro (Wave Metrics). Statistical analyses were conducted with SigmaPlot 12.1 or 12.5 (Systat Software), and differences between the two samples were considered statistically significant if the p-value was < 0.05.

### Morphometric analysis of PC dendrites

Neurobiotin was injected into PCs after electrophysiological analysis, after which the slices were fixed with 4% PFA. After incubation in blocking buffer (5% NGS, 0.5% Triton X-100, 0.1% azide) for 1 h at room temperature, neurobiotin-injected PC dendrites were stained with Alexa 488-conjugated streptavidin (1:1000; Invitrogen) in antibody dilution buffer (5% Block Ace, 5% NGS, 0.1% Triton X-100, 0.1% azide in PBS) overnight at 4 °C. Three-dimensional image stacks were acquired on a confocal microscope (Olympus, FV1000), and PC dendrites were manually traced using Neurolucida-8 software (MicroBrightField). The total dendrite length, total dendrite area, and number of dendritic branches were calculated by Neurolucida Explorer (MicroBrightField). Crossing branch points were calculated from three-dimensional reconstructions as described [10]. Grids with individual squares of 10 × 10 μm were overlaid on top of the *z* projection of the confocal stacks, and randomly selected areas corresponding to a minimum of 20% of all grid squares covering the entire dendritic area of a PC were analysed.

### Analysis of CF synapses in PCs

For the analysis of CF synapses, images were obtained with a confocal microscope (Olympus, FV1000). The molecular layer, which is where PC dendrites reside, was divided into five equal bins, each with a width of 100 μm along the dorsoventral axis, for quantification. The number of VGluT2 puncta in each bin at P60 was counted in sagittal sections. Three control mice and three CTCF-cKO mice were used for quantification experiments. Counts were performed on more than three different regions from each mouse (total 42,019 μm^2^ for control mice, 29,914 μm^2^ for CTCF-cKO mice).

### Electron microscopy analysis

Mice at P60 were deeply anaesthetized and perfused transcardially with 25 mM PBS, followed by perfusion with 1.6% PFA and 3% glutaraldehyde in 0.1 M PB (pH 7.3–7.4) for 12 min. Coronal slices (50 μm thick) were cut using a vibrating microslicer (VT-1000; Leica) in 0.1 M PB. After being washed in 0.1 M PB several times, sections were treated with 1% OsO_4_ in 0.1 M PB for 40 min, stained *en bloc* with 1% uranyl acetate for 40 min, dehydrated with ethanol, and flat-embedded in Durcupan resin (Fluka) [46]. Serial ultrathin sections were prepared at a thickness of 70 nm (Ultracut S; Leica). Images were captured using a transmission electron microscope (TEM1010; JEOL).

### SBF-SEM analysis

The mice were deeply anaesthetized and transcardially perfused with 25 mM PBS followed by 4% PFA with 0.5% glutaraldehyde in 0.1 M PB (pH 7.3–7.4). Then, the cerebellum was removed and postfixed in the same fixative at 4 °C. The brains were sectioned at a thickness of 50 µm on a Vibratome slicer. The slices were postfixed in 4% PFA and 1% glutaraldehyde in 0.1 M cacodylate buffer (pH 7.4) at 4 °C overnight. Slices were cut into small pieces and then treated with 2% OsO_4_ (Nisshin EM, Tokyo, Japan) in 0.1 M cacodylate buffer containing 1.5% K_4_[Fe(CN)_6_] (Nacalai Tesque, Kyoto, Japan), washed four times with cacodylate buffer, and incubated with 1% thiocarbohydrazide (Sigma-Aldrich) for 20 min and then with 2% OsO_4_ for 30 min at room temperature. The slices were then treated with 2% uranyl acetate at 4 °C overnight and stained with Walton's lead aspartate at 60 °C for 30 min. The slices were dehydrated by passing the tissue through an ethanol series (60%, 80%, 90%, 95%, and 100% ethanol) at 4 °C; infiltrated sequentially with acetone dehydrated with a molecular sieve, a 1:1 (v/v) mixture of resin and acetone, and 100% resin; and then embedded in Epon 812 with carbon (Ketjen black) [38]. The specimen-embedding resin was polymerized at 40 °C for 6 h, 50 °C for 12 h, 60 °C for 24 h, and, finally, 70 °C for 2 days. After trimming to the region of interest, the samples were imaged via single electron beams with a Sigma or Merlin field emission-type scanning electron microscope (Carl Zeiss, Munich, Germany) equipped with the 3View system and a backscattered electron detector (Gatan, Pleasanton, CA).

### Statistical analysis

Statistical analyses were performed using GraphPad Prism 7 software (GraphPad Software Inc., San Diego, CA). Comparisons of two samples were performed by unpaired Student’s *t*-test, with Welch’s correction for unequal variances when appropriate. Multiple comparisons were made by two-way analysis of variance (ANOVA) with a Bonferroni post hoc test. The sample size is defined in the figure legends and is also shown as individual dots in the bar graphs.

## Results

### Growth retardation with progressive motor dysfunction in *Grid2*-Cre-mediated CTCF-cKO mice

**Fig. 1:**
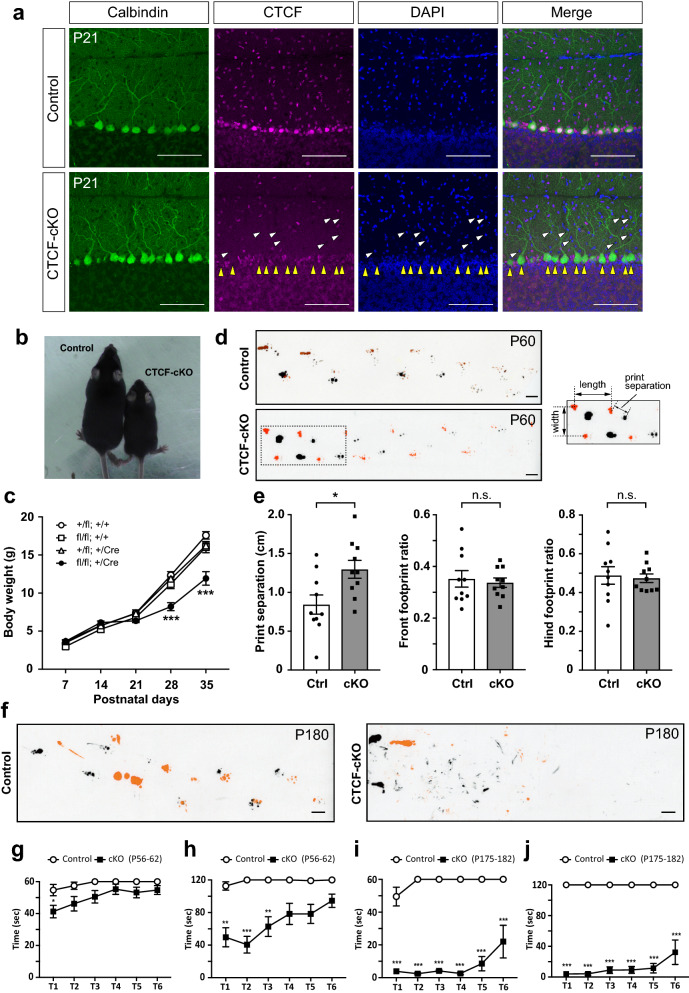
*Grid2*-Cre-mediated CTCF-cKO mice show progressive motor coordination deficits. **a** Confirmation of CTCF deletion by immunohistochemical staining of the cerebellum at P21. Positive anti-CTCF signals (magenta) were not detected in the PCs of CTCF-cKO mice (yellow arrowheads) or some molecular layer neurons (white arrowheads). Scale bars: 100 μm. **b** The body size of CTCF-cKO mice was smaller than that of control littermates. Mice at P30 are shown. **c** Body weight over time from P7 to P35 was measured for each genotype. n = 11 *Ctcf*;*Grid2*-Cre (+/fl; +/+), n = 8 *Ctcf*;*Grid2*-Cre (fl/fl; +/+), n = 9 *Ctcf*;*Grid2*-Cre (+/fl; +/Cre), n = 11 *Ctcf*;*Grid2*-Cre (fl/fl; +/Cre). Significant differences were found between *Ctcf*;*Grid2*-Cre (fl/fl; +/Cre) and other genotypes at P28 and P35. **d** Footprint analysis. Representative footprint patterns of control and CTCF-cKO mice at P60. Black, forepaws. Orange, hindpaws. Print separation and stride length and width are defined as indicated (right panel). Scale bars: 1 cm. **e** Print separation (left), front footprint ratio (width/length of forepaw, centre), and hind footprint ratio (width/length of hidpaw, right). n = 10 (control, cKO). **f** Footprint analysis. Representative footprint patterns of a control and CTCF-cKO mouse at P180. Orange, forepaws. Black, hindpaws. Scale bars: 1 cm. **g–j** Rotarod test. A total of six trials (T1–T6) were conducted for each mouse at P56–62 (**g**, **h**) and P175–182 (**i**, **j**). (**g**, **i**) In one set of trials, rod rotation occurred at a constant rate of 5 rpm over a 60-s period. (**h**, **j**) In a second set, rod rotation accelerated from 0 to 3 rpm over the first 60 s, and the mice were measured for a maximum of 120 s. **g** n = 11 (control), n = 19 (cKO); **h** n = 9 (control), n = 12 (cKO); **i**, **j** n = 6 (control), n = 7 (cKO). n.s., not statistically significant; *p < 0.05, **p < 0.01, ***p < 0.005. Error bars represent the SEM

Immunostaining of the wild-type (WT) mouse cerebellum at postnatal day (P)21 showed that CTCF was most highly expressed in PCs, and expression was higher in the molecular layer cells than in granular layer cells (Fig. 1a). To investigate the role of CTCF in cerebellar PCs, we generated *Grid2*-Cre-dependent CTCF-cKO mice, in which Cre recombinase is predominantly expressed in PCs by P7 and then in a subset of the molecular layer cells [50]. A strong reduction in CTCF was found in CTCF-cKO PCs at P7, but some CTCF remained (Additional file 6: Fig. S1, online resources). By P21, CTCF was not observed in the subset of molecular layer cells and absent in CTCF-cKO PCs at P21 (Fig. 1a).

CTCF-cKO mice were born at the expected Mendelian ratio and exhibited no obvious differences from control littermates during the neonatal and early infantile periods under direct observation. Approximately 3 weeks after birth, CTCF-cKO mice, represented by the genotype *Ctcf*;*Grid2*-Cre (fl/fl; +/Cre), showed obvious differences in appearance with growth retardation and ataxia-like motor abnormalities as compared with mice of other genotypes (Fig. 1b, c). To evaluate the gait and mobility of CTCF-cKO mice, we performed several motor tests. *Ctcf*;*Grid2*-Cre (+/fl; +/+) and *Ctcf*;*Grid2*-Cre (fl/fl; +/+) mice were used as controls. Footprinting analysis showed that print separation, the distance between the front and hind paws, was significantly larger than control front paw and hind paw distances at P60 in CTCF-cKO mice (Fig. 1d, e). In an open-field analysis, CTCF-cKO mice were less mobile than control mice at P50 (Additional file 6: Fig. S2a, online resources).

In addition, we performed walking initiation, beam, platform, pole, and inverted screen tests to examine the motor function of juveniles from P20 to P45. In the walking initiation test, CTCF-cKO mice exhibited a significant increase in the delay before they began walking (Additional file 6: Fig. S2b, online resources). Similar results were observed for the beam test (Additional file 6: Fig. S2c, online resources). In the platform test from P20 to P35 and the pole test from P20 to P25, there were no significant differences between control and CTCF-cKO mice, but the differences became more pronounced during later stages (Additional file 6: Fig. S2d, e, online resources). Thus defects in CTCF in PCs resulted in impaired motor coordination, which might be progressive. In contrast, the differences became less pronounced as age increased in the inverted screen test, a method for evaluating grip strength, for which there were no statistically significant differences between control and CTCF-cKO mice at P45 (Additional file 6: Fig. S2f, online resources).

We continued follow-up observations after P60 to determine if progressive motor dysfunction was occurring in CTCF-cKO mice and noticed that motor dysfunction in these mice became progressively more severe. We attempted a footprint analysis again at P180 but could not track the footprints because the CTCF-cKO mice exhibited very confusing footprint patterns (Additional file 1: Video 1, Additional file 2: Video 2, online resources) as compared with control mice (Fig. 1f). Therefore, we performed a rotarod test to quantify the motor ability of these mice aged P56–62 and P175–182. First, the rotarod test was carried out at a constant rate and at an accelerating rate at P56–62. At a standard constant rate of 5 rpm, CTCF-cKO mice fell from the rotarod within a shorter time period relative to controls during the first trial but exhibited no significant differences as compared with the controls during subsequent trials (Fig. 1g). Under conditions of acceleration from 0 to 3 rpm, CTCF-cKO mice fell from the rotarod sooner during the first three trials when compared with the controls, but significant differences were absent for the last three trials (Fig. 1h). Under these conditions, we tested CTCF-cKO mice on a rotarod at P175–182. The time spent on the rotarod by CTCF-cKO mice at P175–182 decreased markedly during both the constant and accelerating revolution rates as compared with that at P56–62, although the time on the rotarod tended to increase as the trials progressed (Fig. 1i, j). These results indicate that CTCF-cKO mice showed progressively more severe motor dysfunction between P56–62 and P175–182.

### Proximal shift in the climbing fibre (CF) innervation territory on PC dendrites in CTCF-cKO mice

**Fig. 2:**
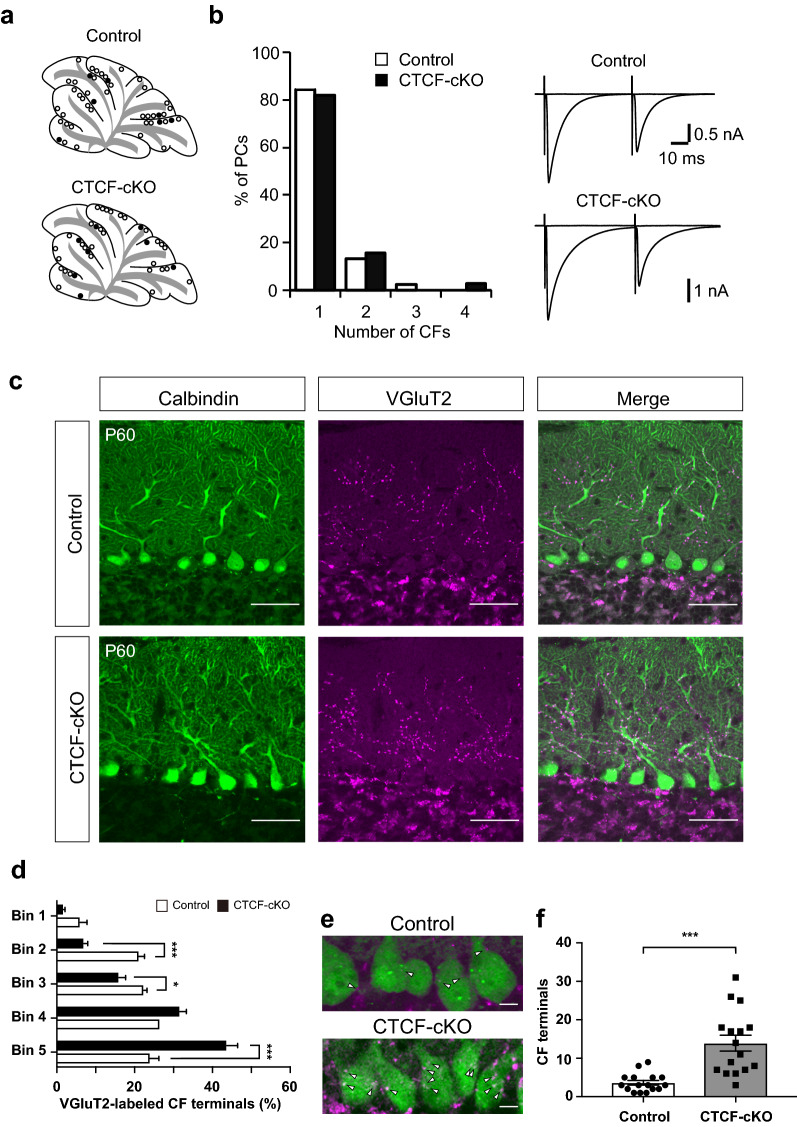
Proximal shift of CF innervation territories in CTCF-cKO mice. **a** Interlobular distribution of recorded PCs in control (upper) and CTCF-cKO (lower) mouse brains. Open and closed circles represent mono-innervated and multiply innervated PCs, respectively. **b** (left) Frequency distribution of the number of CFs innervating individual PCs in control (n = 45 cells, 4 mice) and CTCF-cKO (n = 38 cells, 4 mice) mice aged P47–P59. (right) Representative CF-EPSC traces in a control (upper) and a CTCF-cKO (lower) mouse. Holding potential was − 10 mV. Several traces evoked around the threshold are superimposed. **c** Distribution of CF terminals in the molecular layer in control (upper) and CTCF-cKO (lower) mice at P60. CF terminals and PCs were stained using anti-VGluT2 (magenta) and anti-calbindin (green), respectively. Scale bars: 50 μm. **d** Relative densities of CF terminals in the molecular layer in control (n = 4) and CTCF-cKO (n = 4) mice. The molecular layer was evenly divided into five bins from the bottom to the pia surface, and the number of CF terminals in individual bins was determined (see Additional file 6 Fig. S4g, online resources). **e** Representative images of VGluT2-positive CF terminals (arrowheads) on the PC somata in control (upper) and CTCF-cKO (lower) mice. Scale bars: 10 μm. **f** Number of CF terminals on PC somata. n = 12 (control, cKO). *p < 0.05, ***p < 0.005. Error bars represent the SEM

To assess whether CTCF defects in PCs influence synaptic transmission, we performed whole-cell recording from PCs and examined excitatory postsynaptic currents evoked by CF stimulation (CF-EPSCs) at P47-59 (Fig. 2a). In most PCs in control and CTCF-cKO mice, CF-EPSCs were elicited in an all-or-none manner with a gradual increase in stimulus intensity (Fig. 2b), which suggests that a single CF innervates the PC that was recorded from. The number of CFs innervating individual PCs was not significantly different between control and CTCF-cKO mice (Fig. 2b). We confirmed that CTCF was not deleted in inferior olive neurons in the CTCF-cKO mice (Additional file 6: Fig. S3, online resources). At birth, PCs are innervated by multiple CFs, after which all but one CF are gradually removed, with a single CF innervation becoming established around the third postnatal week in mice [19]. These data suggest that CF synapse elimination proceeds normally in CTCF-cKO mice. The paired-pulse ratio, 10–90% rise time, and decay time constant of CF-EPSCs were normal in CTCF-cKO mice (Additional file 6: Fig. S4a–c, online resources), except for a slight increase in the amplitude of CF-EPSCs (Additional file 6: Fig. S4d, online resources). Although multiply innervating CFs begin with relatively similar synaptic strengths, one CF is strengthened during 1^st^ postnatal week before the start of CF synapse elimination [18, 20]. To evaluate the relative strengths of a single CF among multiple CFs, we analysed the disparity ratio (see materials and methods) in PCs innervated by multiple CFs. We confirmed no differences between control and CTCF-cKO mice (Additional file 6: Fig. S4e, online resources). We also examined parallel fibre-mediated EPSCs (PF-EPSCs); no differences in the paired-pulse ratios of PF-EPSCs were found (Additional file 6: Fig. S4f, online resources). Taken together, excitatory synaptic transmission mediated by CFs and PFs is largely normal in CTCF-cKO mice.

In the adult cerebellum, CFs form synaptic terminals along the proximal parts of PC dendrites. We next investigated CF terminal distribution in the molecular layer by immunohistochemical staining using an antibody against VGluT2, a marker for CF terminals. The molecular layer was evenly divided into five bins from the bottom to the pia surface, and the number of CF terminals in individual bins was counted (Additional file 6: Fig. S4g, online resources). CF terminals were evenly distributed from bins 2 to 5 in control mice; however, their distribution was shifted toward the basal part of the PC dendrites (bin 5) in CTCF-cKO mice (Fig. 2c, d). This trend was observed in all cerebellar lobules (Additional file 6: Fig. S4h, online resources). Whereas the PC somata were largely devoid of CF terminals in the control adult cerebellum, many CF terminals were observed in those of CTCF-cKO mice (Fig. 2e, f). These data suggest that the CF innervation territory undergoes a proximal shift in CTCF-cKO mice.

### Abnormal development and dendrite self-crossing in CTCF-disrupted PCs

**Fig. 3:**
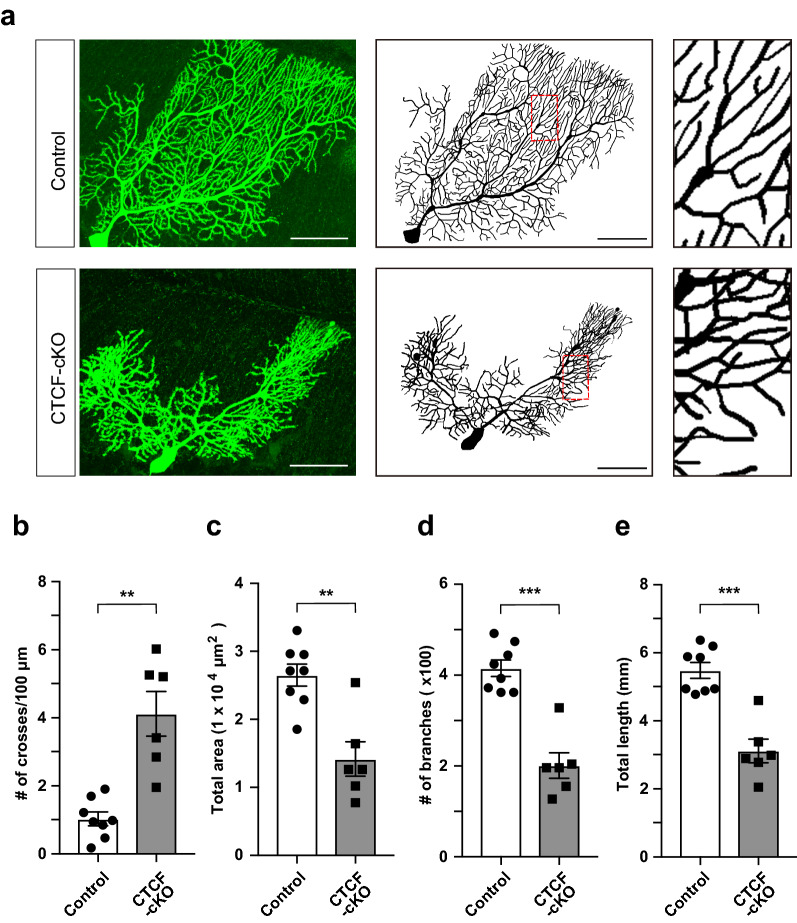
Loss of CTCF causes abnormal dendritic arborization with dendritic crossing in PCs. **a** Neurobiotin staining of a control and CTCF-cKO PC at P47-59 (left) and the resulting traced PCs (middle). A dendrite located in the region of the red box is shown at higher magnification (right) for each PC. Neurobiotin was injected into PCs as part of the patch-clamp recording process. Scale bars: 50 μm. **b**–**e** Quantitative analysis of the number of self-crossing dendrites (**b**), total area (**c**), number of branches (**d**), and total dendritic length (**e**). n = 8 neurons (control), n = 6 neurons (cKO). **p < 0.01, ***p < 0.005. Error bars represent the SEM

Expression of the *cPcdh* gene families *Pcdhα*, *Pcdhβ*, and *Pcdhγ*, and, in particular, stochastically expressed *cPcdh* isoforms, is regulated by CTCF [13, 16, 24]. In addition, PCs of mice with Pcdh deletion (i.e., deletion of Pcdhα, Pcdhγ, or Pcdhα and Pcdhγ) show dendritic self-avoidance defects [26, 32]. We confirmed that expression of stochastically expressed *cPcdh* isoforms such as *Pcdhα12* and *PcdhγA7* was downregulated to undetectable levels in CTCF-cKO PCs at P60 (Additional file 6: Fig. S5, online resources). To examine the contribution of CTCF to dendritic morphology, we injected a neurobiotin tracer into the PCs at P47-59 and then analysed the cells (Fig. 3a). The number of self-crossing dendrites was significantly increased in CTCF-cKO PCs (Fig. 3b). Other parameters, such as total area, the number of dendritic branches, and total length, were significantly decreased in CTCF-cKO cells (Fig. 3c–e). These results indicated that gene regulation by CTCF was important not only for proximal shift in the CF innervation territory but also for dendritic self-avoidance and normal dendritic development in PCs.

### GLB development in PC dendrites of CTCF-cKO mice

**Fig. 4:**
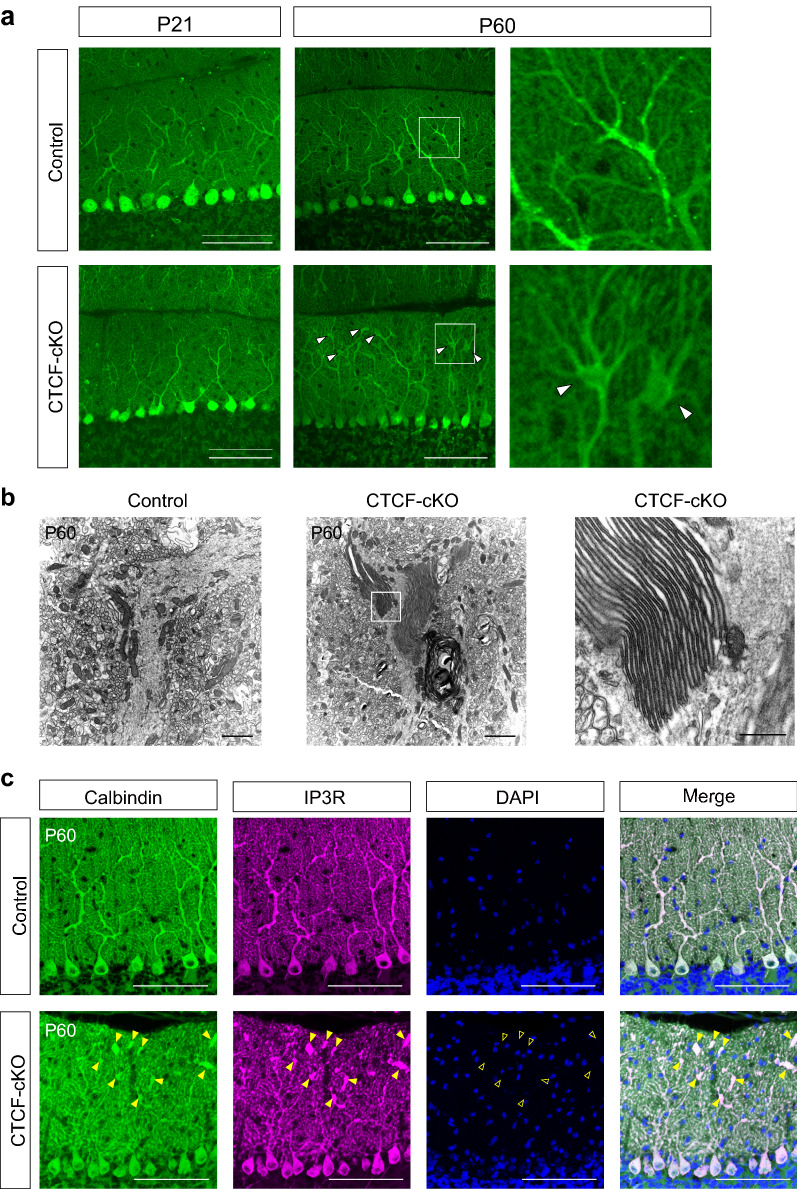
GLBs are found in palm-like dendritic swelling of PC. **a** Anti-calbindin immunohistochemical staining at P21 and P60. Arrowheads indicate palm-like swelling at the dendritic branch points of PCs. Digitally zoomed images (right) correspond to the region in the white box in the middle panels. **b** Electron microscopy analysis at P60. GLBs were found in the PC dendrites of CTCF-cKO mice. A higher-magnification image of the region in the white box in the middle panel is shown (right). **c** Immunohistochemical staining with anti-IP3R (magenta). Whereas both arrowheads and open arrowheads indicate the location of palm-like swelling in PC dendrites, the open arrowheads further indicate that there were no DAPI-positive signals (blue) at those locations. Scale bars: 100 μm (**a**, **c**), 2 μm (**b**, left and middle panel), 500 μm (**b**, right panel)

In addition to the above findings, we found palm-like dendritic swelling that were mostly located at the branch points of PCs in CTCF-cKO at P60 (Fig. 4a). These dendritic swellings were not visible at P21. We next examined the microstructure of these dendritic swellings by electron microscopy. Unexpectedly, we found a peculiar lamellar structure that consisted of stacks of many cisternae oriented in parallel (Fig. 4b; Additional file 6: Fig. S6a, online resources).

Since ~ 40 years ago, very similar microscopic structures, referred to as GLBs, have been reported in the dendrites of PCs in autopsy reports of patients with Werdnig-Hoffmann disease, 13q deletion syndrome, and Krabbe disease [23, 37, 47]. These GLBs have in common eosinophilic staining properties. To evaluate the histochemical properties of palm-like dendritic swelling in CTCF-cKO cells, we carried out haematoxylin and eosin (HE) staining, which showed that these structures were also eosinophilic (Additional file 6: Fig. S6b, online resources). GLBs consist of many cisternae of the endoplasmic reticulum (ER) placed in close apposition [23, 37, 47]. In addition, the inositol 1,4,5-triphosphate receptor (IP3R) is related to the formation of ER cisternal stacks, which are also referred to as organized smooth ER (OSER) [45]. Immunohistochemical analysis revealed that the swollen regions at branch points of CTCF-cKO dendrites were intensely stained for the ER markers KDEL and calnexin (Additional file 6: Fig. S6c, d, online resources). These swollen regions were also strongly stained for IP3R (Fig. 4c). Thus the absence of CTCF was associated with the formation over time of GLBs derived from the smooth ER, which occurred most often at the dendritic branch points of PCs.

We note that the causative gene for Werdnig-Hoffman disease is survival motor neuron 1 (*SMN1*) and that for Krabbe disease is galactocerebrosidase (*GALC*). In situ hybridization analysis showed that the expression of these genes was not significantly influenced by the loss of CTCF in the mouse brain (Additional file 6: Fig. S7, online resources).

Next, to clarify the detailed structure of GLBs, we carried out serial block-face scanning electron microscopy (SBF-SEM) analysis at P60. We analysed both the morphology of GLBs and their interactions with other organelles. In highly magnified SBF-SEM images of PC dendrites in CTCF-cKO cells, the GLBs appeared as stacks and concentric circles of cisternae (Additional file 6: Fig. S8a, online resources). Reslicing of serial images showed that the appearance of concentric circles visible in an *x*–*y* plane was mostly attributable to the curved stack of cisternae clearly observed in the *y*–*z* and *x*–*z* planes (Additional file 6: Fig. S8b, online resources). The stacked cisternae were connected to one another through tubular junctions at the edge of the tubular smooth ER (Additional file 6: Fig. S8c, online resources). The GLBs often had close contact with mitochondria (Additional file 6: Fig. S8d, online resources).

### Disappearance of most PCs with GLBs from the cerebellum

**Fig. 5:**
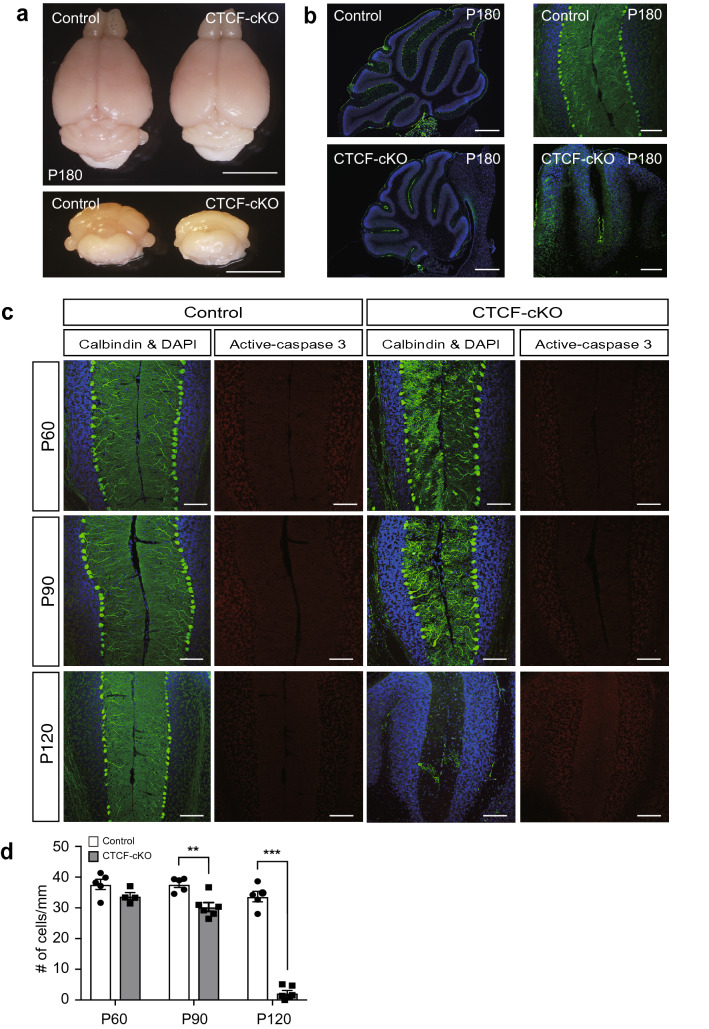
Loss of PCs in CTCF-cKO mice. **a** typical picture of the whole brains of control and CTCF-cKO mice at P180. The size of the cerebellum in CTCF-cKO mice was smaller than that in control mice. **b** Sagittal sections of the cerebellum stained with anti-calbindin (green) and counterstained with DAPI (blue) at P180. The left panel shows that CTCF-cKO mice had smaller cerebellums than control mice. The right panel shows none of the calbindin-positive PCs in CTCF-cKO mice. **c** Temporal analysis of PC loss. The cerebellum was stained with anti-calbindin (green) and anti-active caspase-3 (red) and counterstained with DAPI (blue). PCs were dramatically lost from P90 to P120 in CTCF-cKO mice. We observed no staining differences for active caspase-3 between control and CTCF-cKO mice. **d** Quantification of the number of PCs. n > 4 at each stage for each genotype. **p < 0.01, ***p < 0.005. Error bars represent the SEM. Scale bars: 5 mm (**a**), 500 μm (**b**, left panel), 100 μm (**b**, right panel and **c**)

Previous reports of human diseases that involve GLBs showed that pronounced neurodegeneration was also present [23, 37, 47]. The severe motor dysfunction in CTCF-cKO mice between P60 and P180 (Fig. 1f–j) may be due to neuronal cell loss. To assess this possibility, we analysed the cerebellum at P180. The size of the cerebellum of CTCF-cKO mice was obviously smaller than that of control mice (Fig. 5a). We performed immunohistochemical observation of PCs and found few PCs in the cerebellums of CTCF-cKO mice at P180 (Fig. 5b). Accordingly, the cerebellums were sampled at P60, P90, and P120 to determine when PC loss was occurring. Temporal analysis revealed that PC loss had already started at P90, and the number of PCs had dramatically decreased at P120; then, almost all PCs were lost by at least P180 (Fig. 5b–d). We next attempted to detect apoptotic cell death using an antibody against active caspase-3 at P60, P90, and P120, but we did not detect significant differences in PCs between mice in the control and CTCF-cKO groups (Fig. 5c). In contrast, active caspase-3-positive signals were increased in the molecular layer especially at P120. These results indicated that deletion of CTCF in PCs induced progressive motor dysfunction with dramatic PC loss from P90 to P120, which might have been due to nonapoptotic cell death.

**Fig. 6:**
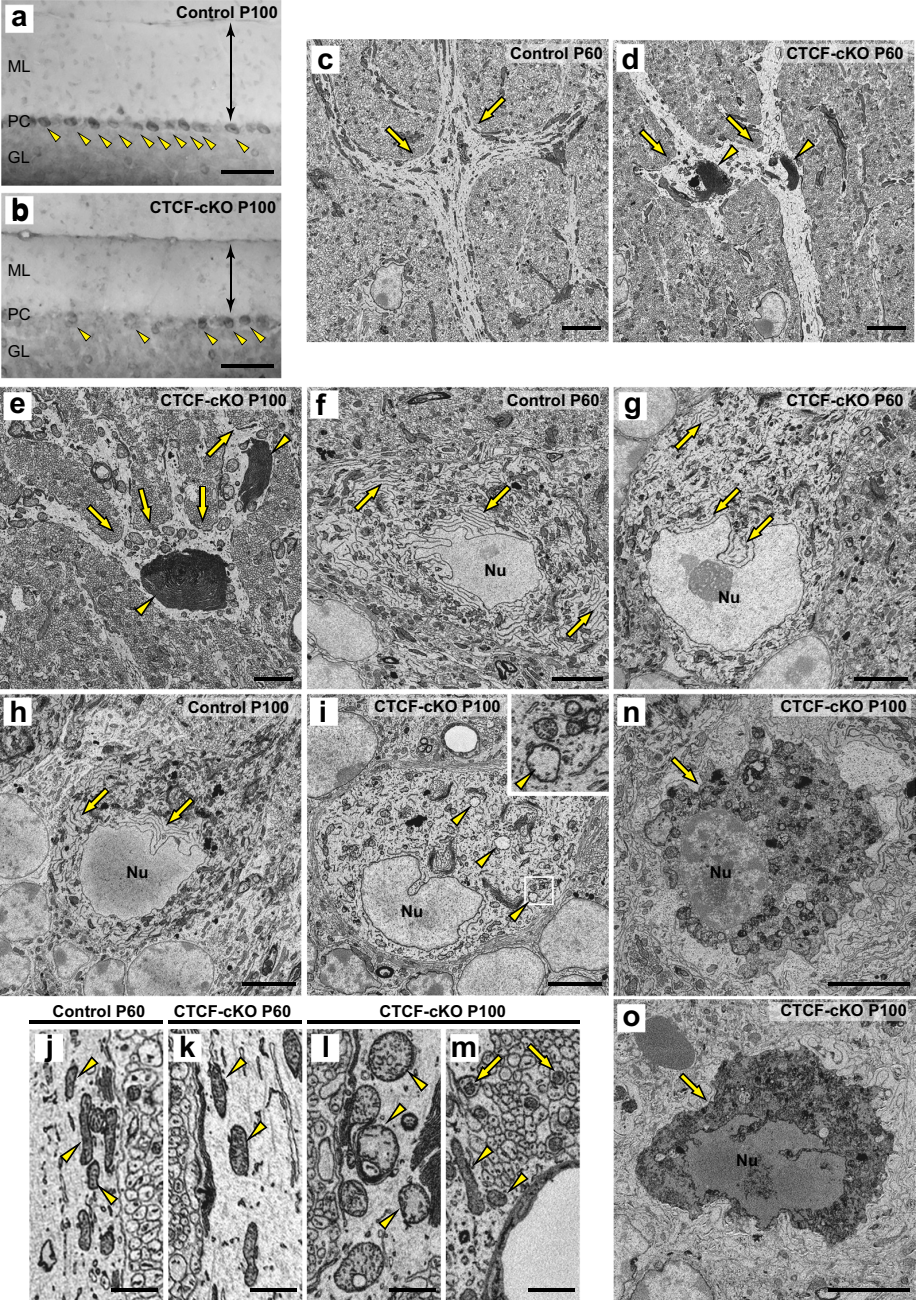
Temporal analysis of morphological changes in organelles in PCs by SBF-SEM. **a**, **b** Toluidine blue staining at P100. Arrowheads indicate PCs. Double-headed arrow indicates the molecular layer (ML). The Purkinje cell (PC) layer and granular layer (GL) are also shown. **c**–**e** Typical SBF-SEM images of PC dendrites in control mice at P60 (**c**) and CTCF-cKO mice at P60 (**d**) and P100 (**e**). Arrows indicate the dendritic branch points, where GLBs typically form only in CTCF-cKO mice (d, e, arrowheads). **f**–**i** Morphological changes in the nucleus (Nu) and around the nucleus. Nuclei and Nissl bodies (arrows) are shown in control mice at P60 (**f**) and P100 (**h**) and in CTCF-cKO mice at P60 (**g**) and P100 (**i**). Arrowheads in (**i**) indicate swollen mitochondria with reduced cristae. The area marked with a white box is magnified in the inset. **j**–**l** Morphological changes in mitochondria in PC dendrites. Mitochondria in PC dendrites are shown for control mice at P60 (**j**) and in CTCF-cKO mice at P60 (**k**) and P100 (**l**). Arrowheads indicate thin normal mitochondria (**j**), slightly swollen mitochondria (**k**), and swollen mitochondria (**l**). **m** Normal mitochondria of parallel fibres (arrows) and processes of Bergmann glia (arrowheads) surrounding a blood vessel (BV) are shown in a CTCF-cKO mouse at P100. **n**, **o** PCs in CTCF-cKO mice at P100 that show cellular debris (arrow) without nuclear fragmentation. Scale bars: 100 μm (**a**, **b**), 5 μm (**c**–**i**, **n**, **o**), and 1 μm (**j**–**m**)

To clarify temporal changes in GLBs and other organelles in PCs, we carried out SBF-SEM analysis of mice at P60 and P100. Staining with toluidine blue showed a thinner molecular layer and a decreased number of PCs in CTCF-cKO mice at P100 (Fig. 6a, b). This indicated that the PCs were in the process of cell death at this time point. GLBs were observed in PC dendrites of CTCF-cKO mice at both P60 and P100, whereas dendrites of control mice had no GLBs (Fig. 6c–e). One of the most noticeable changes in CTCF-cKO mice from P60 to P100 was the change in the size of GLBs, which were obviously larger at P100 than at P60 (Fig. 6d, e; Additional file 3: Video 3, Additional file 4: Video 4, online resources). The ER around the nuclei of PCs was short, intermittent, and generally diminished in CTCF-cKO cells at P60 as compared with that in control cells, in which the ER was well developed (Fig. 6f, g). Moreover, the ER was almost absent around the nuclei in CTCF-cKO cells at P100 (Fig. 6h, i). We also observed morphological changes in mitochondria that are associated with neuronal viability and neurodegeneration [11, 12]. Mitochondria in PC dendrites were thin in control cells and slightly swollen in CTCF-cKO cells at P60 (Fig. 6j, k), whereas mitochondrial swelling and the partial absence of cristae were prominent in CTCF-cKO cells at P100 (Fig. 6i, l). In contrast, no mitochondrial swelling was observed in parallel fibres or processes of Bergmann glia surrounding blood vessels (Fig. 6m). We note that the fine structure of nuclei was maintained at both P60 and P100 in PCs from CTCF-cKO mice, although there were many mitochondria with abnormal morphology characterized by prominent swelling and decreased cristae at P100 (Fig. 6h, i). Furthermore, some CTCF-cKO PCs exhibited plasma membrane rupture, and the nuclei were surrounded by cellular debris, but we could not find nuclear fragmentation at P100 (Fig. 6n, o; Additional file 5: Video 5, online resources). This observation indicates that cell death occurred due to nonapoptotic mechanisms and is consistent with the present immunohistochemical results (Fig. 5c).

## Discussion

Here we investigated the role of CTCF in PCs and found characteristic phenotypes (Additional file 6: Fig. S9, online resources**)**. CTCF-cKO mice showed growth retardation and progressive motor dysfunction. They also showed a proximal shift in the CF innervation territory and defects in PC dendrite self-avoidance. Furthermore, we found GLBs in the dendrites of PCs at P60 in the knockout mice. A temporal analysis of these cells revealed that GLBs developed gradually from P60 to P100. Over the same time period, we noted a gradual reduction in the ER, until this structure was almost absent around PC nuclei by P100. By P180, PCs with GLBs absent in the cerebellum.

The cerebellum, in which PCs are the only neuronal cells that send inhibitory projections to the deep cerebellar and vestibular nuclei, has important roles in body movement and balance. In the present study, we showed that CTCF-cKO mice exhibit motor dysfunction by P60. In the PCs of these mice, we found aberrant distribution of CF terminals, dendritic self-avoidance defects, and dendritic developmental impairment by P60. The arborization of dendrites is important in characterizing different types of neurons, and dendritic configuration influences the formation of neural circuits. In addition, dendrites from the same neuron are generally arranged so that they do not cross each other, which is called self-avoidance. *cPcdh,* especially isoforms that are stochastically expressed, is regulated by the higher-order chromosomal structure established by CTCF, and cPcdh is a key molecule for self-avoidance based on its direct and indirect genetic effects in PCs. Self-avoidance defects are associated with the deletion of Pcdhγ [32]. Deletion of Pcdhα also has a similar effect on dendritic self-avoidance, and Pcdhα and Pcdhγ double mutants show a more severe phenotype of dendritic self-avoidance and reduced dendritic area than single mutants [26]. As an indirect effect, defects in self-avoidance along with abnormal dendritic arborization are also associated with deletion of Dnmt3b, which regulates stochastic *cPcdh* expression by controlling the methylation state of the *cPcdh* promoter regions [48]. Thus, the absence of dendritic self-avoidance and arborization in CTCF-cKO mice is consistent with the expected results, although the influence of other molecules under the control of CTCF cannot be ruled out.

GLBs have been reported in patients with Werdnig-Hoffman disease, 13q deletion syndrome, and Krabbe disease [23, 37, 47]. These reports were all case reports based on autopsies. GLBs have also been reported in miniature poodle pups as a case report [3]. In these cases, the GLBs were eosinophilic, were composed of stacks of many ER cisternae oriented in parallel, and were localized to the dendrites of PCs. In contrast, lamellar bodies, which are smaller than GLBs, have been observed under hypoxic conditions, in electric fields, and under conditions associated with fixation [17, 22, 51]. Because of very limited reports of GLBs, it has been difficult to determine whether GLBs represent complete pathological changes or including artificially induced changes. In this study, we identified eosinophilic inclusion bodies that had formed in the dendrites of PCs and were composed of many stacked ER cisternae in CTCF-cKO mice. These results are consistent with the previous observations of GLBs and showed that GLBs represent a pathological alteration of PCs. In addition, IP3R was also highly concentrated at the swollen regions. IP3R is highly expressed in PCs and is localized throughout the ER membrane, with a particularly high concentration in the ER cisternal stacks [45]. Our results suggested that the high concentration of IP3R contributes to the formation of GLBs in the PC dendrites. The responsible gene loci, however, are different between this mouse model and human disease. The loci for the diseases for which GLBs have been reported are as follows: Werdnig-Hoffman disease, survival motor neuron 1 (*SMN1*) on 5q13.2; Krabbe disease, galactocerebrosidase (*GALC*) on 14q31.3; and 13q deletion syndrome, [46, XY, del (13) (q22q31)]. In the case of the miniature poodle pups, the locus is unknown. *CTCF,* for which GLBs were confirmed in this study, is located on 16q22.1 in the human genome, and expression of neither *Smn1* nor *Galc* was significantly influenced by the loss of CTCF in mice. These results indicate that GLBs are a common neuropathological feature across a background of different genetic alterations.

After P60, more severe defects in motor function are thought to be due to loss of neuronal cells. Previous studies have suggested that neurodegeneration may develop by the late stage of GLB formation [3, 23, 37]. In this study, we observed that the formation of GLBs was associated with increasing age. The dendritic swellings associated with the location of GLBs were not visible at P21, but these same regions had become remarkably swollen by P60. Neuronal loss was not clearly observed at P60, which was consistent with the absence of remarkable changes that would suggest a threat to cell survival based on the structure of mitochondria and the ER around the nuclei of PCs. In the CTCF-cKO mice, the loss of PCs started gradually around P90, and almost all PCs were lost by P120. At P100, as cell death was progressing, the size of GLBs in the PC dendrites was obviously larger than that at P60, the ER was almost absent around the nuclei, and the mitochondria had swollen markedly and showed a drastic decrease in cristae. It seems that the ER from the perikaryon had shifted to the dendritic region, where it contributed to GLB formation. A major factor in the death of these PCs may be the absence of the ER around the cell nucleus that is associated with GLB development. Nonetheless, our immunohistochemical and SBF-SEM analyses indicated that the cell death of PCs was intrinsically mediated by nonapoptotic death mechanisms, although these mechanisms are currently unknown. Our temporal observations strongly support the view that PC loss develops during the late stage of GLB organization.

## Conclusion

In conclusion, our study is the first to report, experimentally and reproducibly, that GLBs were formed in PCs in associated with the loss of CTCF. The causative genetic background for GLBs, however, can vary. Thus, our results indicate that GLBs are a common neuropathological feature due to different causative genes and suggest that the formation of GLBs may occur in patients with other diseases that are accompanied by motor dysfunction. In addition, we confirmed that the PCs with GLBs eventually succumbed to cell death. Neuronal degeneration and loss of PCs have been reported in previous human diseases with GLBs [23, 37, 47]. We can now address the questions concerning which molecules under the control of CTCF promote the formation of GLBs, why GLBs are more likely to accumulate in dendrites of PCs, and whether there is a direct relationship between GLB accumulation and cell death.

## Supplementary Information

**Additional file 1.** Video 1. Typical gait of control mice at P180.

**Additional file 2.** Video 2. Typical gait of CTCF-cKO mice at P180.

**Additional file 3.** Video 3. SBF-SEM images of GLB in a CTCF-cKO mouse at P100.

**Additional file 4.** Video 4. SBF-SEM images of GLB in a CTCF-cKO mouse at P100.

**Additional file 5.** Video 5. SBF-SEM images of PC in a CTCF-cKO mouse at P100.

**Additional file 6.** Supplementary figures and legends.
