# Supplementary material for: CTCF loss induces giant lamellar bodies in Purkinje cell dendrites
Source: Acta Neuropathol Commun. 2022 Nov 29;10:172. doi: 10.1186/s40478-022-01478-6 (PMC9706876; doi:10.1186/s40478-022-01478-6)
Supplement: Supplementary file 6 — Additional file 6. Supplementary figures and legends. [file 40478_2022_1478_MOESM6_ESM.pdf]

Supplementary figures

CTCF loss induces giant lamellar bodies in Purkinje cell dendrites

Teruyoshi Hirayama[1, 2, *], Yuuki Kadooka[1], Etsuko Tarusawa[1], Sei Saitoh[3, 4], Hisako Nakayama[5, 6], Natsumi Hoshino[1], Soichiro Nakama[2], Takahiro Fukuishi[2], Yudai Kawanishi[2], Hiroki Umeshima[2], Koichi Tomita[2], Yumiko Yoshimura[7, 8], Niels Galjart[9], Kouichi Hashimoto[6], Nobuhiko Ohno[10, 11], Takeshi Yagi[1]

[1]KOKORO-Biology Group, Laboratories for Integrated Biology, Graduate School of Frontier Biosciences, Osaka University, Suita 565-0871, Japan.

[2]Department of Anatomy and Developmental Neurobiology, Tokushima University Graduate School of Medical Sciences, 3-18-15 Kuramoto-cho, Tokushima 770-8503, Japan.

[3]Section of Electron Microscopy, Supportive Center for Brain Research, National Institute for Physiological Sciences, Okazaki 444-8787, Japan.

[4]Department of Anatomy II and Cell Biology, Fujita Health University School of Medicine, 1-98 Dengakubo, Kutsukake-cho, Toyoake 470-1192, Japan.

[5]Department of Physiology, Division of Neurophysiology, School of Medicine, Tokyo Women's Medical University, Tokyo 162-8666, Japan.

[6]Department of Neurophysiology, Graduate School of Biomedical and Health Sciences, Hiroshima University, 1-2-3 Kasumi, Minami-ku, Hiroshima 734-8551, Japan.

[7]Section of Visual Information Processing, National Institute for Physiological Sciences, National Institutes of Natural Sciences, Okazaki, Aichi 444-8585, Japan.

[8]Department of Physiological Sciences, The Graduate University for Advanced Studies, Okazaki, Aichi 444-8585, Japan.

[9]Department of Cell Biology, Erasmus University Medical Center, P.O. Box 2040, 3000 CA Rotterdam, the Netherlands.

[10] Division of Ultrastructural Research, National Institute for Physiological Sciences, Okazaki 444-8585, Japan.

[11]Department of Anatomy, Division of Histology and Cell Biology, Jichi Medical University, Shimotsuke 329-0498, Japan.

*Correspondence: hirayama@tokushima-u.ac.jp

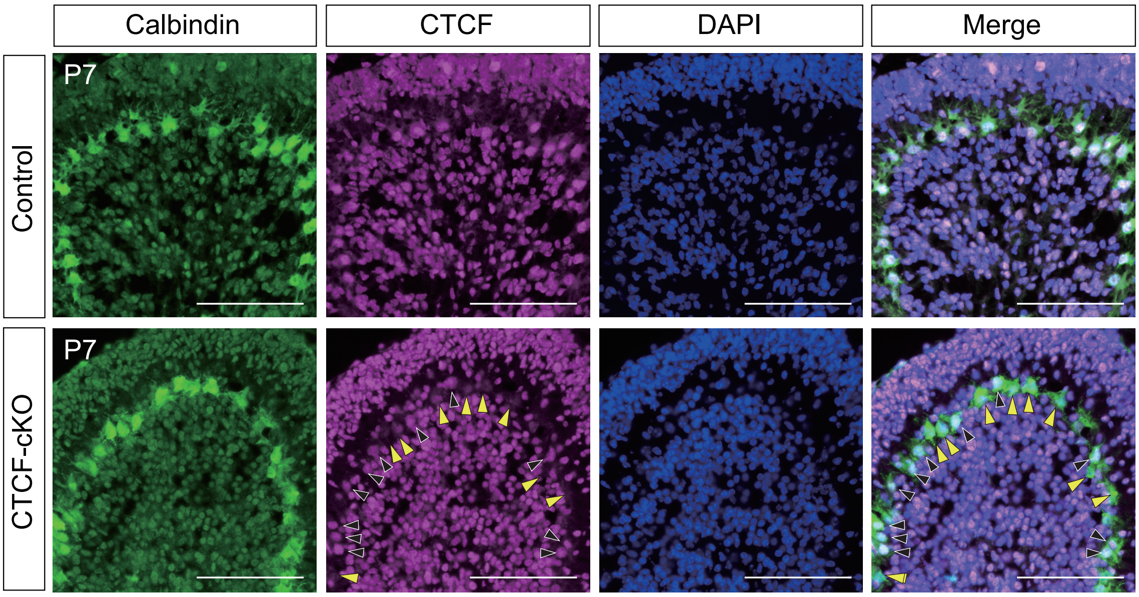

Supplementary Fig. 1 Confirmation of CTCF deletion by immunohistochemical staining of the cerebellum at P7. Positive anti-CTCF signals (magenta) were markedly decreased or were not detected in some PCs (yellow arrowheads), whereas other PCs did express this protein (black arrowheads) in CTCF-cKO mice. At this time point, CTCF expression was similar in the molecular layer cells of both control and CTCF-cKO mice. Scale bars: 100 μm.

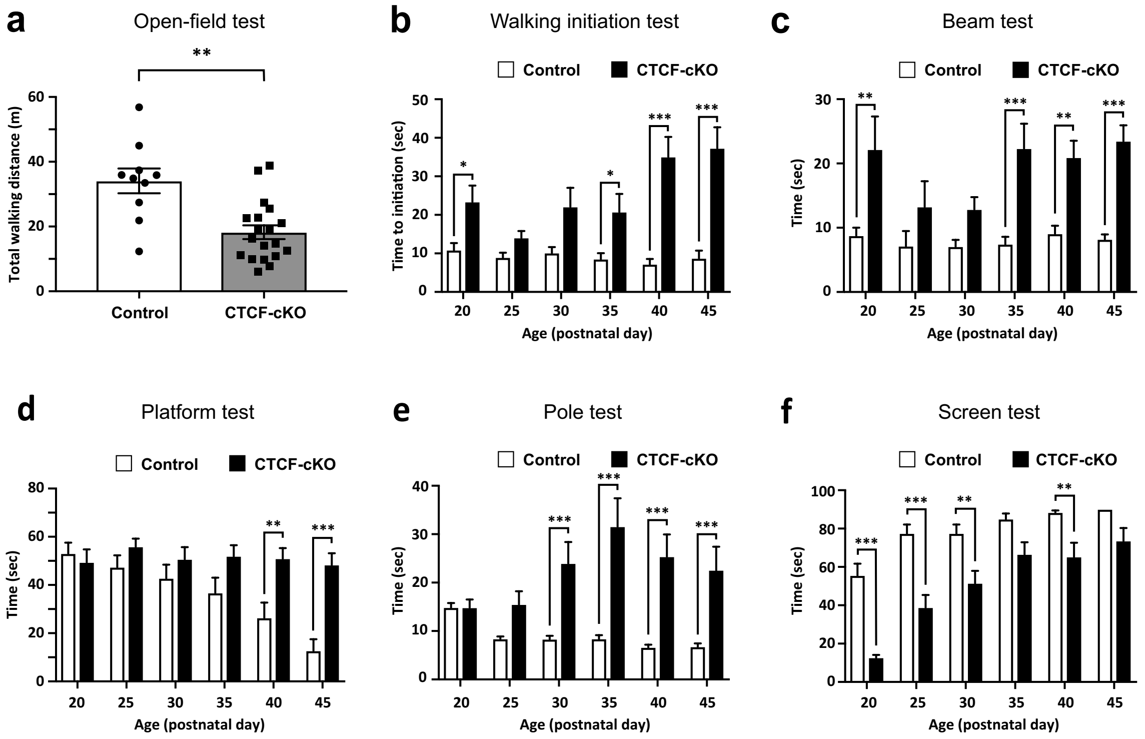

Supplementary Fig. 2 Behavioural studies. **a** Open-field test. Average walking distance for control and CTCF-cKO (cKO) mice at P50 over 10 min. n = 10 (control), n = 19 (cKO). **b** Walking initiation test. n = 18 (control), n = 15 (cKO). **c** Beam test. The time was measured for each mouse to traverse a beam that was 700 mm long. n = 16 (control), n = 13 (cKO). **d** Platform test. The time was measured for each mouse to remain on an elevated, square platform. n = 16 (control, cKO). **e** Pole test. A mouse was placed with its head upwards on top of a vertical metal rod (diameter, 8 mm; height, 70 cm), and the time was measured for each mouse to take to descend to the floor. n =19 (control), n = 15 (cKO). **f** Screen test. The time was measured for each mouse to remain on upside down on the wire mesh grid screen. n = 27 (control), n = 16 (cKO). *p < 0.05, **p < 0.01, ***p < 0.005. Error bars represent the SEM.

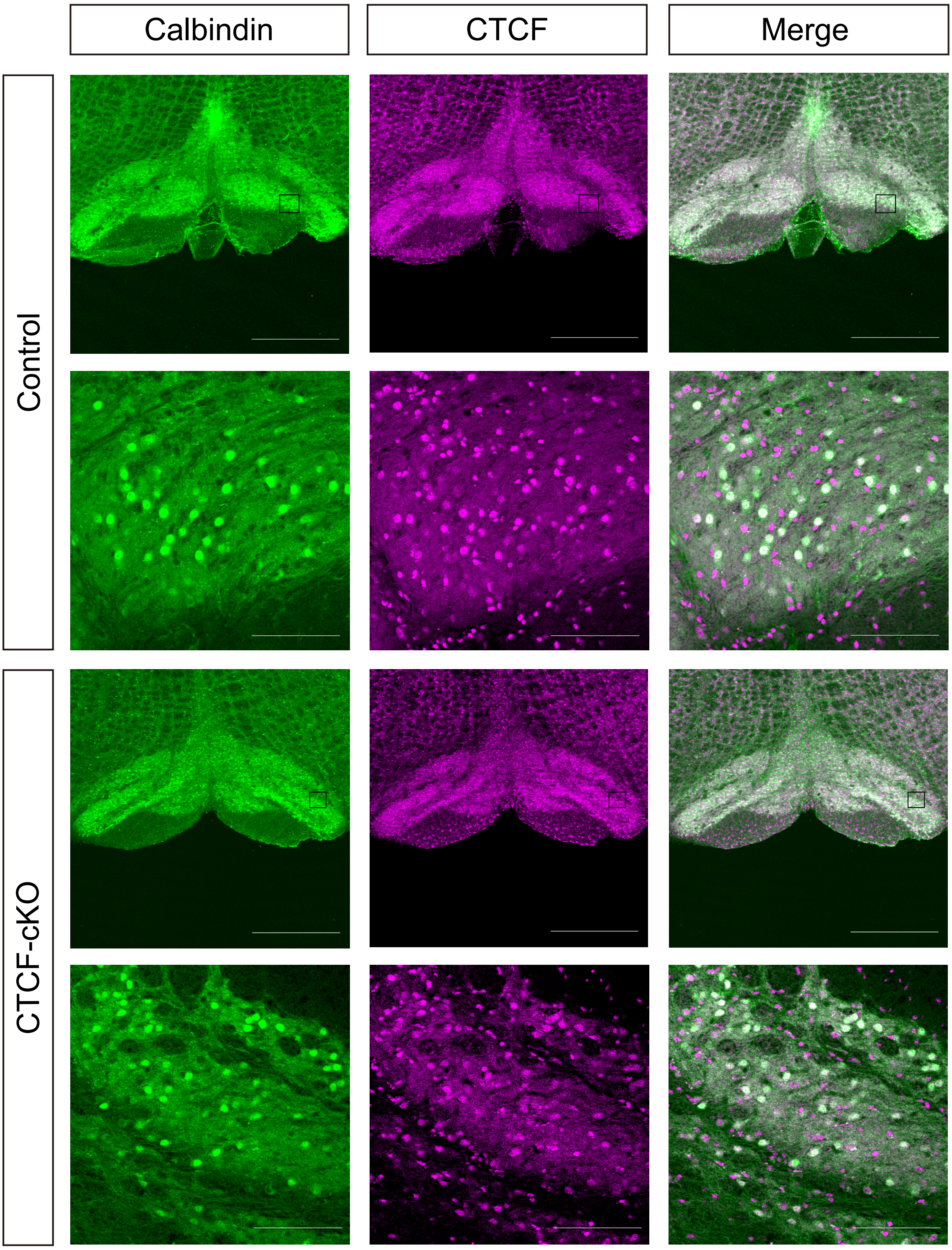

Supplementary Fig. 3 Immunohistochemical staining of the inferior olive at P60. Inferior olive neurons were stained with anti-calbindin (green). CTCF expression was detected by anti-CTCF (magenta). The lower picture of each genotype shows high-

magnification images of the boxed regions in each upper image. Scale bars: 300 μm (upper panel) and 150 μm (lower panel).

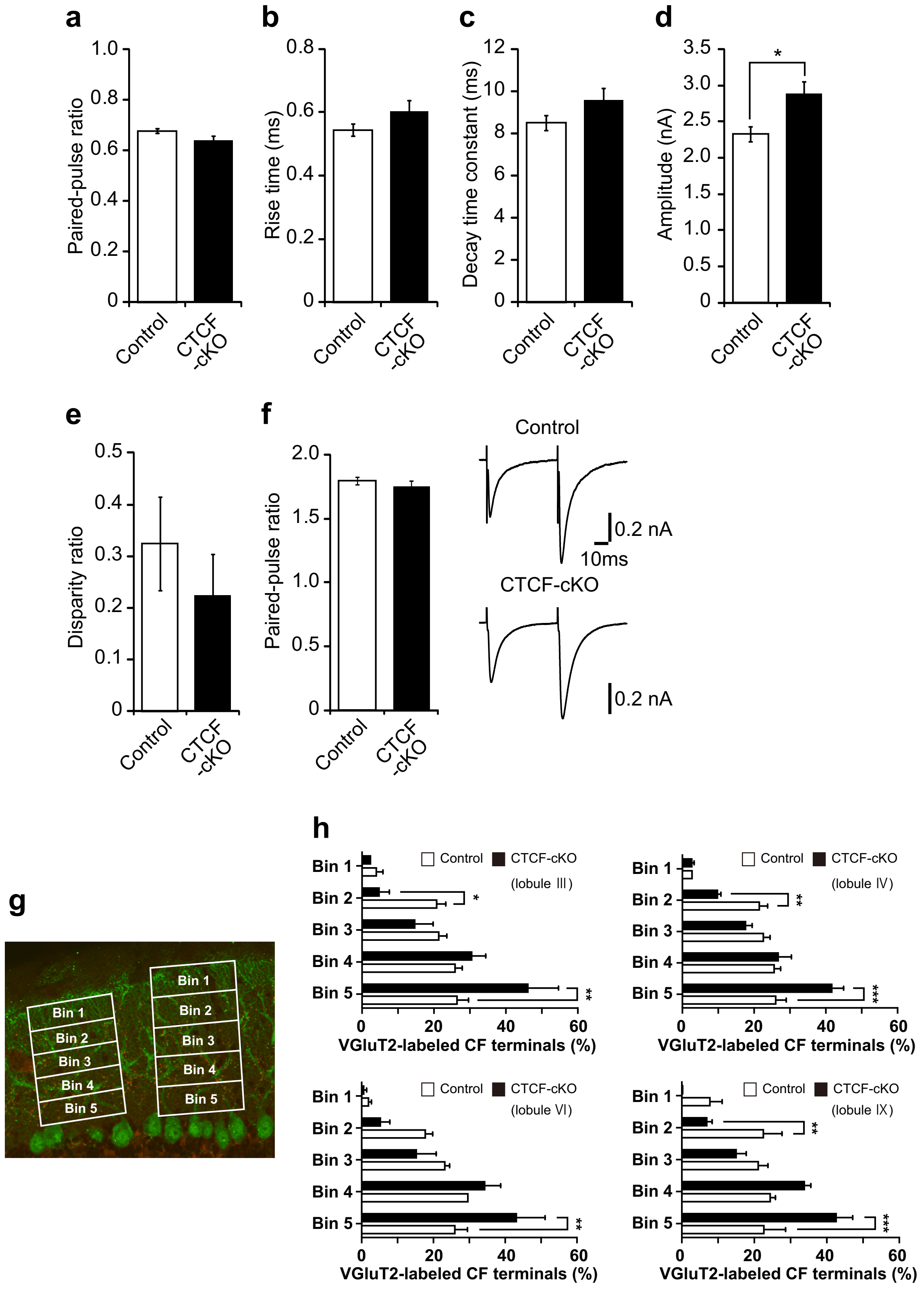

Supplementary Fig. 4 Kinetical properties of CF- and PF-EPSCs, and morphological evidence for the proximal shift of CF innervation territories in CTCF-cKO mice. **a-d**

Electrophysiological properties of EPSCs elicited by a single innervating CF. Paired-pulse ratio (a), rise time (b), decay time constant (c), and amplitude (d). n = 37 cells (control), 29 cells (cKO) each from 4 mice. **e** Disparity ratio of CF-derived EPSCs for PCs that were innervated by two or more CFs (control, n = 7 cells from 4 mice; cKO, n = 6 cells from 4 mice). **f** Average paired-pulse ratio of PF-derived EPSCs (left) and a typical example of PF-EPSC traces from control and CTCF-cKO cells (right). n = 39 cells (control), 38 cells (cKO) each from 4 mice. **g** PC dendrites of the molecular layer were divided into five equal bins (100 μm wide) along the dorsoventral axis for quantification. **h** The results of each examined cerebellar lobule. *$p < 0.05$, **$p < 0.01$, ***$p < 0.005$. Error bars represent the SEM.

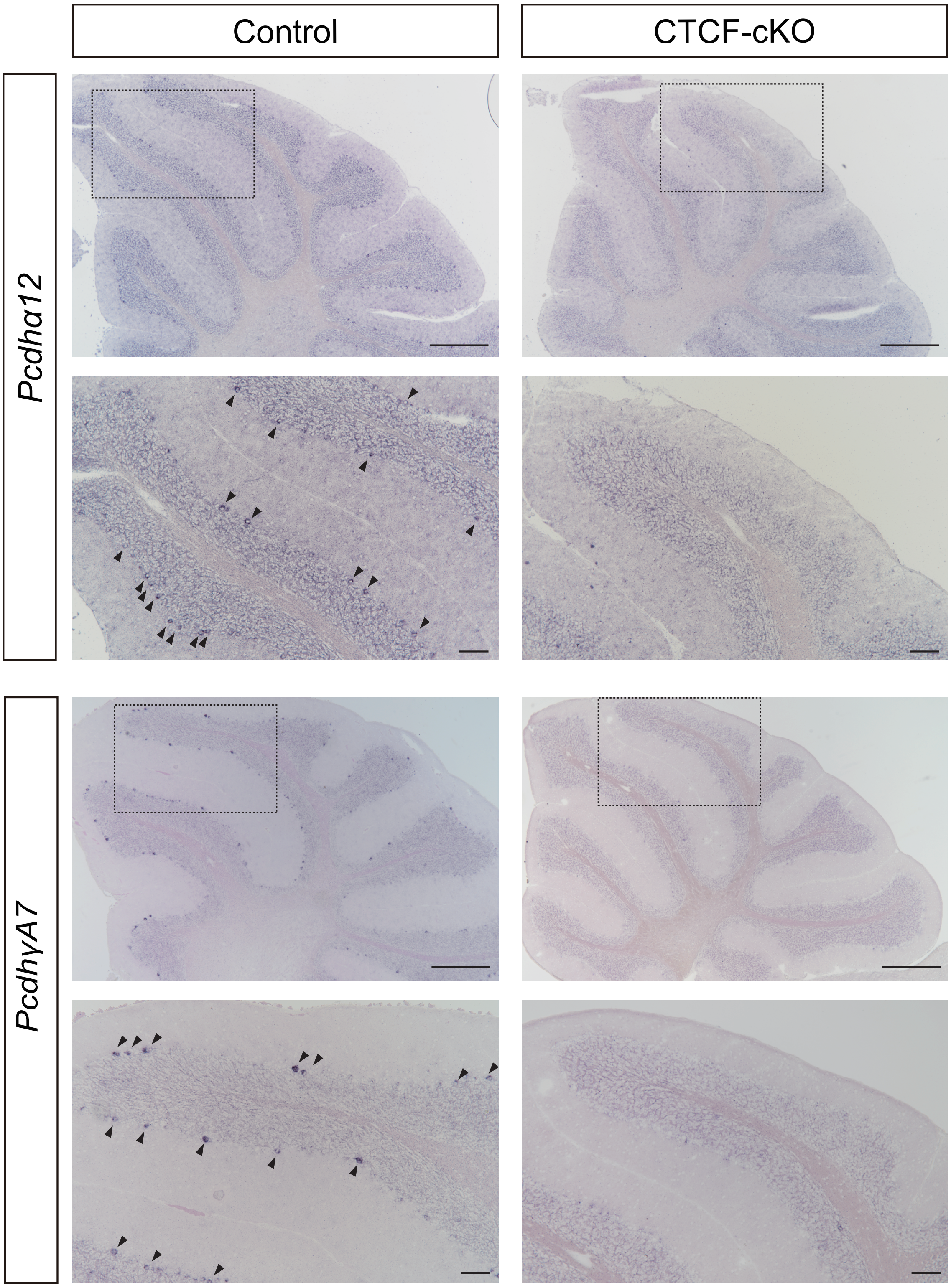

Supplementary Fig. 5 In situ hybridization analysis of *Pcdha12* and *PcdhγA7* expression in PCs in control and CTCF-cKO mice at P60. Example of *Pcdha12* and *PcdhγA7* expression in the cerebellum. The lower image of each genotype shows the boxed region

of the corresponding upper image at higher magnification. Arrowheads indicate positive signals in PCs. Scale bars: 500 μm (upper panel), 100 μm (lower panel).

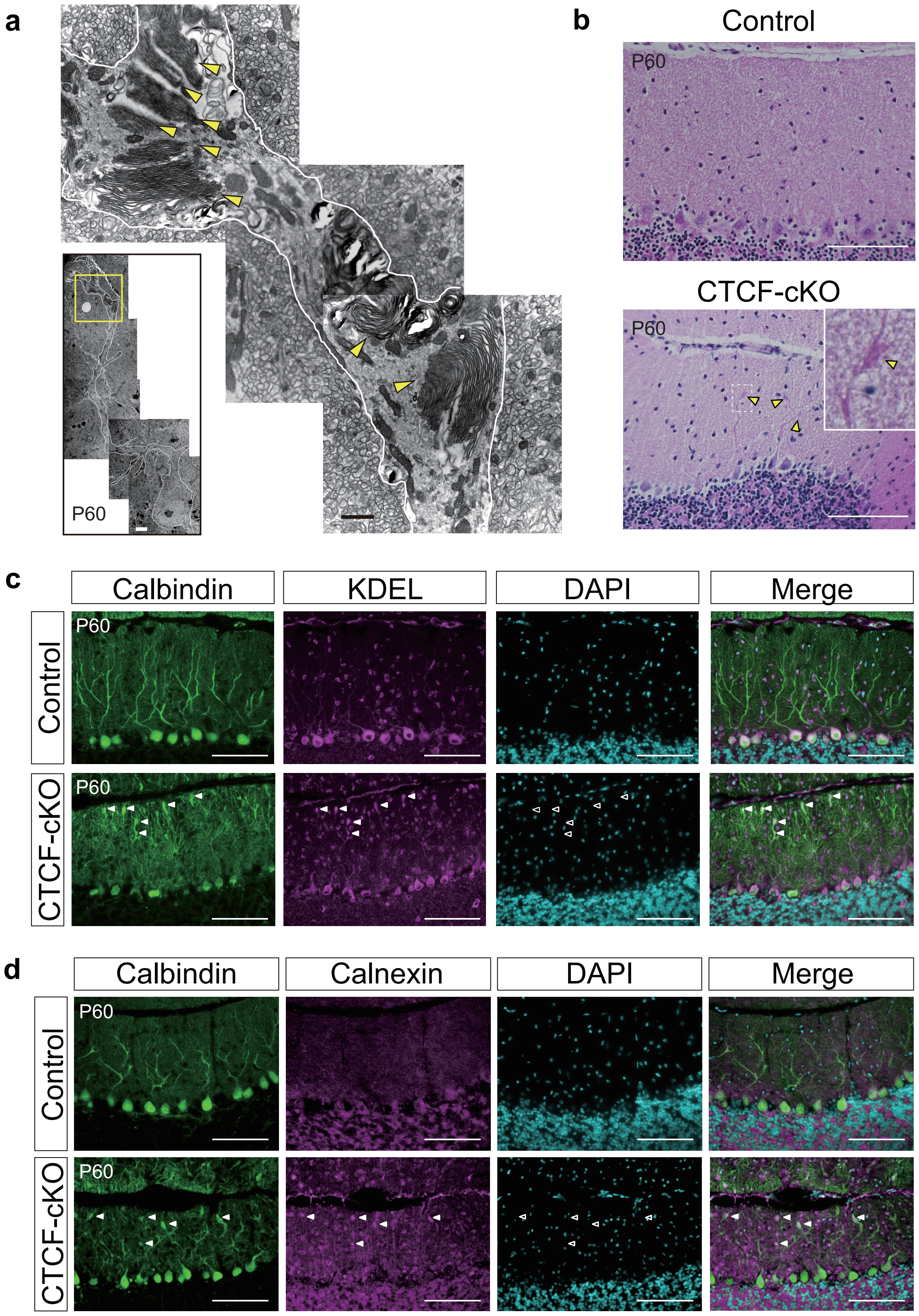

a
P60
b
Control
P60
CTCF-cKO
P60
c
Calbindin
KDEL
DAPI
Merge
Control
P60
CTCF-cKO
P60
d
Calbindin
Calnexin
DAPI
Merge
Control
P60
CTCF-cKO
P60

Supplementary Fig. 6 Localization and properties of GLBs. **a** A typical image of GLBs in a PC dendrite from a CTCF-cKO mouse at P60 acquired by electron microscopy. The lower left image shows the dendrites of a PC from the soma (located in the lower right) to the location of the GLBs (yellow box). The upper right image shows the region in the yellow box at higher magnification. Yellow arrowheads indicate GLBs. White lines indicate the dendritic boundaries. **b** HE staining of dendritic branch points of PCs in control and CTCF-cKO mice at P60. The presence of eosinophilic staining (arrowheads) of palm-like swelling is shown. The area marked with a broken-line is magnified in the inset. **c** Immunohistochemical staining with anti-KDEL (magenta) as a marker of the ER. Arrowheads and open arrowheads indicate the locations of palm-like swelling in PC dendrites. In addition, open arrowheads indicate that there were no DAPI-positive signals (blue) at those locations. **d** Immunohistochemical staining with anti-calnexin (magenta) as a marker of the ER. Arrowheads and open arrowheads indicate the location of palm-like swelling in PC dendrites. In addition, open arrowheads indicate that there were no DAPI-positive signals (blue) at those locations. Scale bars: 5 μm (a, lower left), 1 μm (a, upper right), 100 μm (b, c).

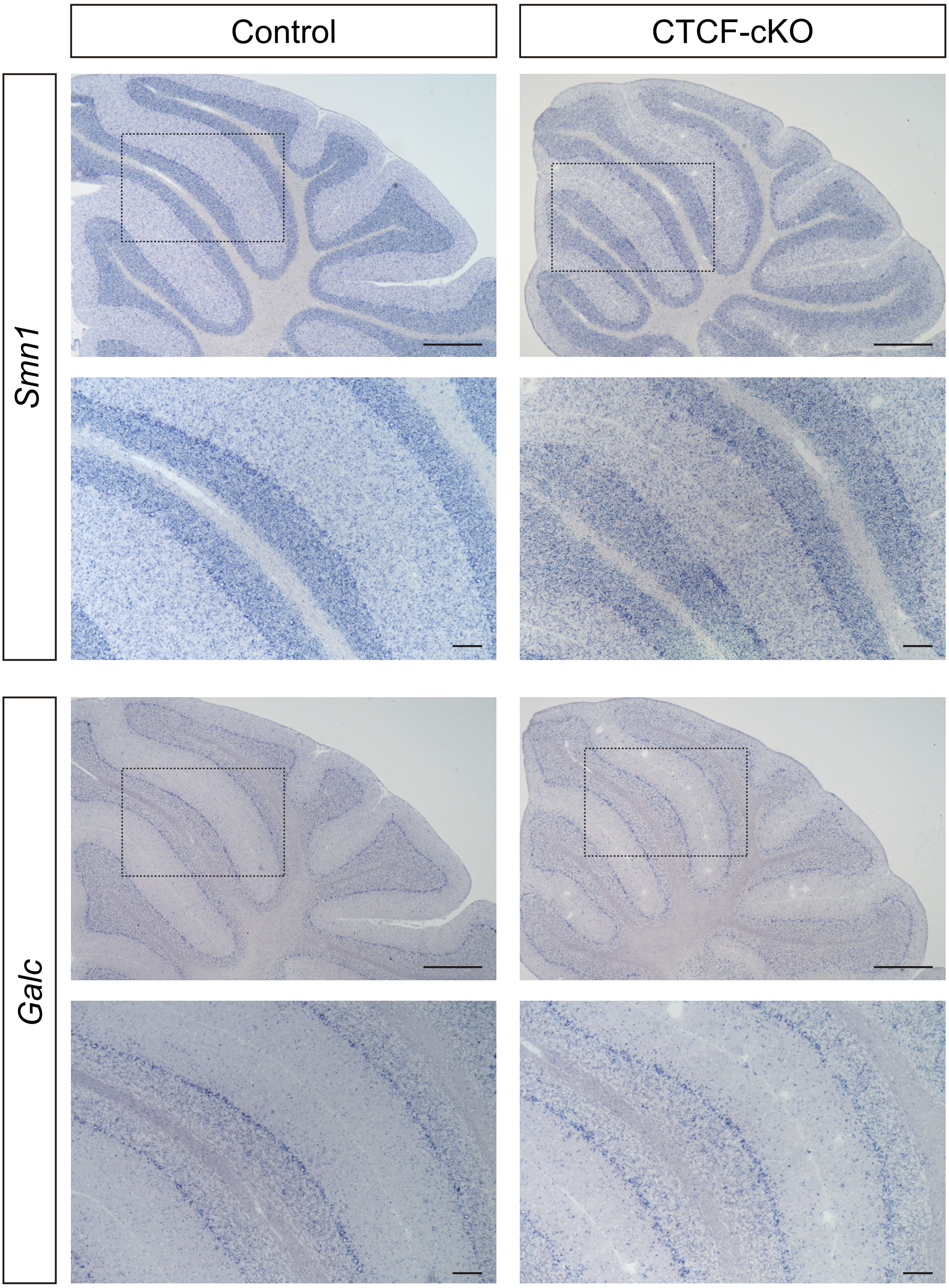

Supplementary Fig. 7 In situ hybridization analysis of *Smn1* and *Galc* expression in PCs in control and CTCF-cKO mice at P60. Example of *Smn1* and *Galc* expression in the cerebellum. The lower image of each genotype shows the boxed region of the

corresponding upper image at higher magnification. Scale bars: 500 μm (upper panel), 100 μm (lower panel).

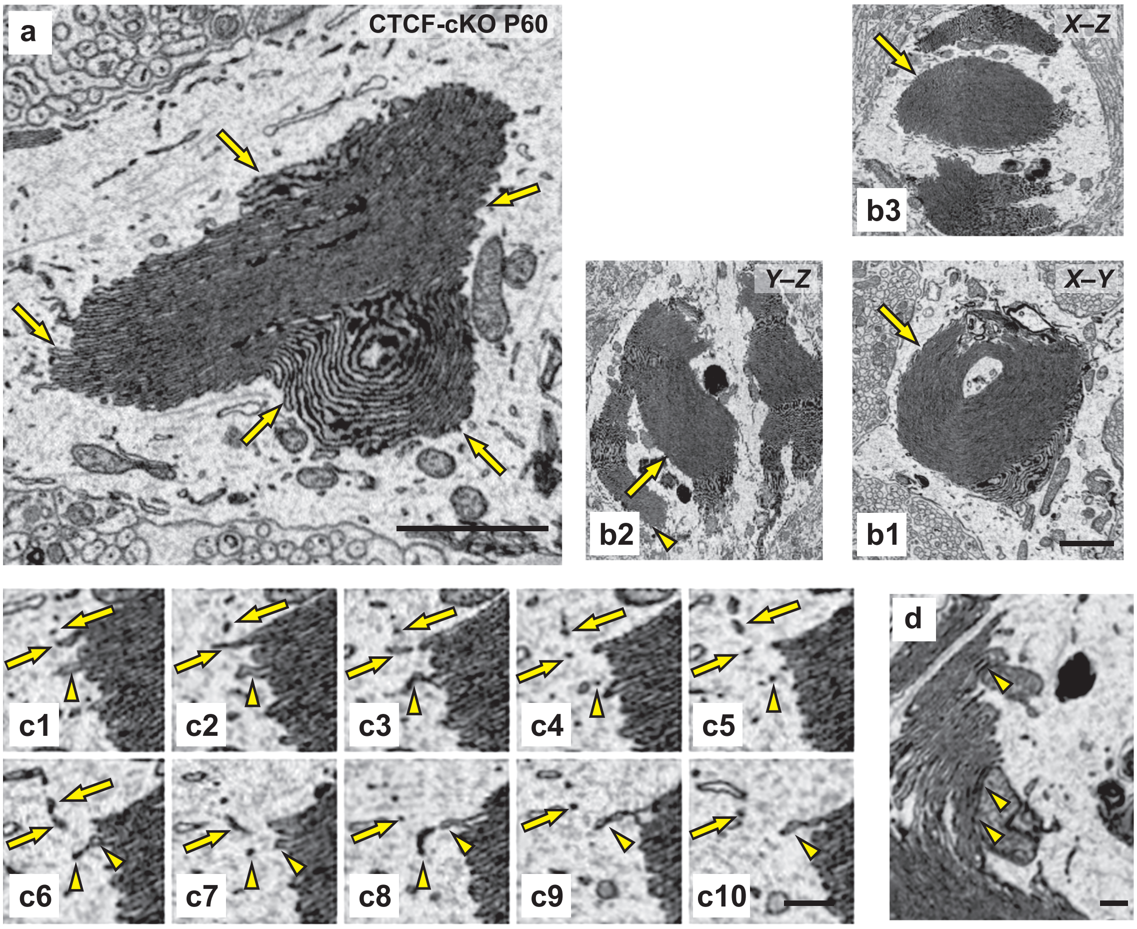

Supplementary Fig. 8 Morphology of GLBs and their interactions with other organelles. **a** SBF-SEM image of a PC dendrite in CTCF-cKO mice. The GLBs are indicated (arrows). Reslicing of serial images was carried out in the *x*–*y* plane (b1, arrow), *y*–*z* plane (b2, arrow), and *x*–*z* plane (b3, arrow). Tubular junctions at the edges of the cisternae (c1–10, arrowheads) and tubular smooth ER (c1–10, arrows) are shown. The GLBs and mitochondria (d, arrowheads) are shown. Scale bars: 1 μm (a, b), 200 nm (c, d).

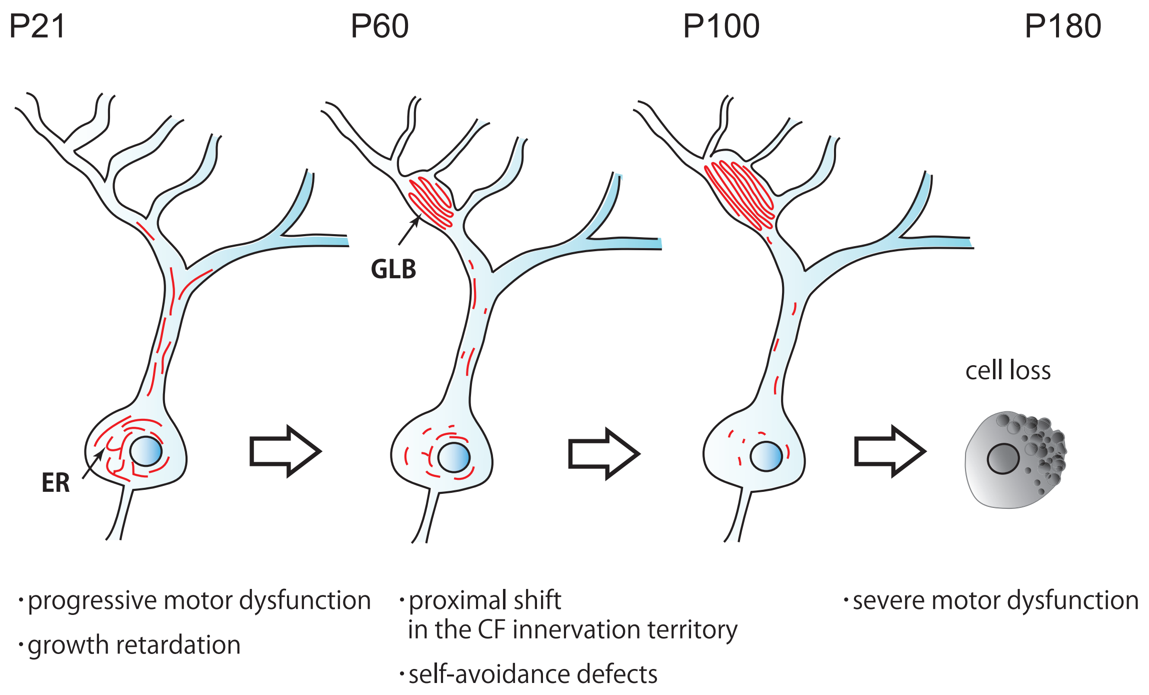

Supplementary Fig. 9 A summary graphic of phenotypes observed in PCs from CTCF-cKO mice at the indicated ages.
